# Screening chest radiography: results from a Greek cross-sectional survey

**DOI:** 10.1186/1471-2458-6-113

**Published:** 2006-04-29

**Authors:** Konstantinos Kamposioras, Giovanni Casazza, Davide Mauri, Velisarios Lakiotis, Ivan Cortinovis, Apostolos Xilomenos, Christina Peponi, Vassilis Golfinopoulos, Athanasios Milousis, Dimitrios Kakaridis, Georgios Zacharias, Ioanna Karathanasi, Georgios Ferentinos, Anastasios Proiskos

**Affiliations:** 1 Panhellenic Association for Continual Medical Research (PACMeR) Sections of Public-Health 28, Karolou st, 10438 Athens, Greece; 2 Istituto di Statistica Medica e Biometria, Università degli studi di Milano Via Venezian 1, CAP 20133 Milano, Italy

## Abstract

**Background:**

Public health authorities worldwide discourage the use of chest radiography as a screening modality, as the diagnostic performance of chest radiography does not justify its application for screening and may even be harmful, since people with false positive results may experience anxiety and concern. Despite the accumulated evidence, various reports suggest that primary care physicians throughout the world still prescribe chest radiography for screening. We therefore set out to index the use of chest radiography for screening purposes among the healthy adult population and to analyze its relationship with possible trigger factors.

**Methods:**

The study was designed as a cross-sectional survey. Five thousand four hundred and ninety-nine healthy adults, coming from 26 Greek provinces were surveyed for screening practice habits in the nationwide anticancer study. Data were obtained for the use of screening chest radiography. Impact of age, gender, tobacco exposure, family history positive for malignancies and professional-risk for lung diseases was further analyzed.

**Results:**

we found that 20% (n = 1099) of the surveyed individuals underwent chest radiography for screening purposes for at least one time during the previous three years. Among those, 24% do so with a frequency equal or higher than once yearly, and 48% with a frequency equal or higher than every three years. Screening for chest radiography was more commonly adopted among males (OR 1.130, 95% CI 0.988–1.292), pensioners (OR 1.319, CI 1.093–1.593) and individuals with a positive family history for lung cancer (OR 1.251, CI 0.988–1.583). Multivariate analysis confirmed these results.

**Conclusion:**

Despite formal recommendations, chest radiography for screening purposes was a common practice among the analyzed sample of Greek adults. This practice is of questionable value since the positive predictive value of chest radiography is low. The implementation of even a relatively inexpensive imaging study on a national scale would greatly burden health economics and the workload of radiology departments.

## Background

Chest radiography has a long tradition in medical care, however its prescription for screening purposes among healthy individuals is discouraged by public health authorities [1-5]. In fact, due to the low prevalence of tuberculosis in developed countries and the incapability to modify lung cancer-specific mortality, the use of chest radiography as a screening tool is not effective.

The diagnostic performance of chest radiography does not justify its application for screening neither in the general population nor in "high risk" groups like smokers or people with a family history of lung cancer [6,7]. Screening chest radiography is not considered effective, it does not have a high yield, and false positive exams result in additional and unnecessary medical tests, associated economic costs, and patient anxiety and stress [6,7].

Despite the accumulated evidence and the clear guidelines, various reports suggest that primary care physicians throughout the world still prescribe chest radiography for screening both in the general population and in selected "high risk" subgroups [8-16]. Consequently, screening chest radiography may represent a major problem that harms screenees' health, and burdens public-health economics and radiology departments' activities.

Nevertheless, since the proportion of physicians believing in and recommending a screening test may consistently differ from the proportion of healthy individuals undergoing the test (still dependent on patients' will), the negative impact of screening chest radiography on health and economics may be only speculated. Little is in fact known in peer-reviewed literature about how chest radiography for screening purposes is practiced among the general healthy adult population [16-18].

We therefore tried to evaluate the rate of screening chest radiography practice among a large sample of Greek healthy adults. Furthermore, we analyzed the resulting chest radiography screening practice for the impact of professional risk for lung diseases, family history of cancer and smoking practice.

## Methods

### PACMeR_02 trial

This study is part of a large ongoing survey on cancer screening and preventive practice in Greece, which is organized by PACMeR (Panhellenic Association for continual Medical Research), and has the purpose to reveal the current rate of cancer screening among the Greek adult population, to evidence possible barriers to early diagnosis of cancer and to analyze over-practice events and possible sources of worthless costs. For the project, PACMeR physicians had dedicatedly prepared two medical questionnaires (one for male and one for female) for face-to-face interviews that were employed during the research program. The exact phrasing of the chest radiography questions used is provided in the supplementary note for the facilitation of the peer-review process.

The project was ethically approved by PACMeR's Scientific Committee (protocol number 08_020720) and conformed to the ethical guidelines of the 1975 Declaration of Helsinki. A written informed consent form was obtained from all the participants before completing the study questionnaire and the data retrieved were analyzed in anonymous and codified form.

### Population and data extraction

**Figure 1:**
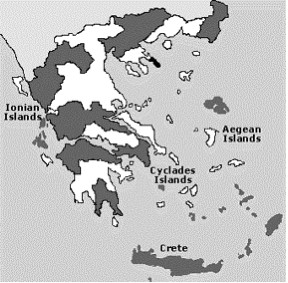
Map of Greece: areas involved in the study (grey).

The study population was composed of a nationwide convenience sample: adults bringing or visiting their relatives while getting healthcare in Hospitals and Health Centers of 26 Greek provinces (Fig. 1). Most populated Greek areas were involved in the study, including more than 80% of the Greek population. Five thousand four hundred and ninety-nine individuals (2948 female, 2551 male, age range 21–97) entered the study and answered the questionnaires during a face-to-face interview between 2000 and 2004.

Ninety-two physicians employed in primary care activities were involved in the study, 87 of them as interviewers, and five as data managers and quality control personnel. Data storing was assured by SESy, a dedicated database [19,20] tailored to population-based cross-sectional surveys for cancer prevention and screening assessment.

Data were extracted for overall chest radiography practice. For each individual we retrieved the chronological period that elapsed from the last chest radiography and the cause for which chest radiography was performed. We further evaluated the proportion of individuals who assessed that they underwent chest radiogram last time for screening purposes. For people who performed it within three years we still analyzed the frequency by which they underwent the test.

### Definition

Since the diagnostic performance of chest radiography does not justify its application in any screening setting, we considered chest radiography being done for screening purposes in any of the following situations: 1) periodic health examination (conducted at regular intervals, e.g. yearly); 2) check-up visit (requested by individuals who do not undergo health examination at regular intervals); 3) chest radiography in asymptomatic individuals due to patients' will; 4) regulatory reasons (driving license, health certificate etc.).

### Subgroup analysis

We analyzed the rate of screening chest radiography by the following parameters: age (<45, 45–64, 65–74 and >75 years old), professional category (pensioners, professions at risk for lung diseases and other professions), cancer family history, smoking activity (no smokers, smokers, ex smokers), number of daily cigarettes smoked (<10, 10–20, 20–30, 30–40, >40, no smokers), duration of tobacco exposure (<10, 10–20, 20–30, 30–40, >40 years).

### Statistical analysis

In order to evidence population subgroups at higher probability of undergoing screening chest radiography, we performed univariate and multivariate analysis. Only subjects for whom there were data about the time elapsed from last chest radiography and about the reason for which they underwent chest radiograms, were considered (n = 5282).

Individuals entering the analysis were therefore divided in:

(1) Subjects at higher probability of undergoing screening chest radiography: people who performed chest radiography for screening purposes (regulatory reason excluded) within the last three years (n = 1080);

(2) Subjects at lower probability of undergoing screening chest radiography exposure: individuals who underwent chest radiography for any other reason (than screening) at any time and those who underwent chest radiograms for screening purposes but more than 3 years had elapsed (n = 4202).

Univariate analysis was used in order to examine the association between over-practice and all subgroups previously defined. Multiple logistic regression analysis was performed to analyze the relationship between over-practice and some relevant covariates of interest: sex, age, professional category, smoke and family history of lung cancer. All independent variables were taken as categorical, dichotomized where appropriate. We used SAS statistical package, version 8.2 [21], for analyses at 95% confidence intervals.

## Results

### Population

The characteristics of the individuals involved in the study are reported in table 1. The mean age of individuals who entered the study was 60.35 years. There was a significant difference in the age distribution of female and male individuals involved in the survey (χ32
 MathType@MTEF@5@5@+=feaafiart1ev1aaatCvAUfKttLearuWrP9MDH5MBPbIqV92AaeXatLxBI9gBaebbnrfifHhDYfgasaacH8akY=wiFfYdH8Gipec8Eeeu0xXdbba9frFj0=OqFfea0dXdd9vqai=hGuQ8kuc9pgc9s8qqaq=dirpe0xb9q8qiLsFr0=vr0=vr0dc8meaabaqaciaacaGaaeqabaqabeGadaaakeaaiiGacqWFhpWydaqhaaWcbaGaeG4mamdabaGaeGOmaidaaaaa@307D@ = 332.89 p < 0.001) largely driven by the higher proportion of women included in the age group < 45 years old (14.3% for women vs 2.3% for men). The mean age of male individuals involved in the study was consistently higher than the mean age of women: 63.4 years old (standard error 0.19) versus 57.7 years old (standard error 0.21) (Table 1).

### Chest radiography patterns

76.6% of the population analyzed (n = 4212) referred that they underwent at least one chest radiogram during their life; 29.5% (n = 1622) assessed that they underwent it last time for screening purposes; 43.4% (n = 2385) performed it in out-patient basis for medical reasons; and 3.7% (n = 205) underwent it in in-patient basis.

Among people who underwent chest radiography for screening purposes, (Table 2) we found that 19.98% (1099 individuals; 537 males and 562 females) did so within a three-year period. Among those (information available for 936 out of 1099), 24.15% (n = 226) underwent it with a frequency equal or higher than once yearly and 47.97% (n = 449) with a frequency equal or higher than every three years. Details on frequencies are reported in table 3.

### Subgroup analysis

Univariate analyses evidenced that the risk of screening radiogram performance was statistically higher among pensioners (OR 1.319, 95%CI 1.093–1.593). Trends to higher chest X-ray practice were still found among individuals of male gender (OR 1.130, 95% CI 0.988–1.292) and those with a family history positive for lung cancer (OR 1.251, 95% CI 0.988–1.583), although these trends were not statistically significant. Interestingly people with professions at risk for lung diseases showed lower probability to undergo screening chest radiograms (OR 0.846, 95% CI 0.713–1.003). Chest radiography performance for screening purposes was not influenced by age, tobacco consumption, and family history positive for malignancies (Table 4).

Multivariate analysis confirmed the results obtained by univariate analysis. Pensioners have a higher probability of undergoing a screening chest x-ray exam (OR 1.277, 95% CI 1.041–1.568); and trends for male gender (OR 1.188, 95% CI 1.013–1.393), and professions at risk for lung diseases (OR 0.808, 95% CI 0.675–0.968) became statistically significant (Table 5).

## Discussion

Screening tests are generally harmful and only in selected cases their benefit outweighs potential harms [22]. In two systematic reviews of older randomized trials there was no evidence supporting the use of chest radiography for lung cancer screening [6,7]. If anything, in these reviews screening with chest radiography was associated with increased lung cancer mortality [7], although this finding is consistent with over-diagnosis bias, given that overall mortality was not affected. Health hazards are not related to radiation exposure, since the delivered dose is very low [23]; they rather stem from the additional diagnostic and/or therapeutic interventions during further evaluation of false positive findings [6]. Indicatively, the proportion of abnormal chest x-ray findings ranges between 3–10% [24,25] with a rate of false positive results ranging from 40–60% [7]. Thus, the implementation of even a relatively inexpensive imaging study on a massive scale would greatly burden health economics and the workload of radiology departments.

Despite the available evidence and recommendations, physicians throughout the world still prescribe chest radiography for screening purposes [8-16]. Little is known in peer-reviewed literature about how chest radiography is practiced for screening purposes among the general healthy adult population. Based on our review of the current literature, only three studies have been published since 1995 [16-18], but all these studies present major limitations. In the study of Woodward (1996) the "perceptions of 452 Canadian physicians about the extent to which patients in their practices obtained screening chest radiography at regular intervals" were investigated [17]. In the study of Hutchison (1998) the proportion of chest radiograms recommended by 62 Canadian physicians during 246 unannounced "standard patients" was evaluated [18]. However no data had been reported in these two studies about the real application of the test among the underlying populations. In the third study (1995), 3281 patients' charts were audited from medical archives of 60 physicians, and data were further abstracted for screening chest radiography practices [16]. Still in this case the information should be considered incomplete since we do not know anything about the proportion of patients who performed the test due to their own will (opportunistic screening), or prescription by another physician.

This is therefore the first study indexing the impact of screening chest radiography habit among a population subgroup. Practice of chest radiograms for screening purposes was common among the examined sample of Greek adults: 20% underwent it for at least one time during the previous three years and among these, 48% declared to perform it with a frequency equal or higher than once every three years.

Interestingly, in logistic regression analyses the high-utilization rates were not strongly driven by smoking practice (smokers versus no smokers), as previously hypothesized [26]. Moreover, people with professions at risk for lung disease also showed lower high-utilization trends. Individuals at major risk for over-screening chest radiograms were male subjects, pensioners and individuals with a family history positive for lung cancer.

The retrieved rates of screening chest radiography should not surprise. In a recent Greek survey of 211 physicians, 88% declared to recommend chest radiography for early diagnosis procedures: 78% prescribed it during usual check-up visit, and 77% recommend it for cancer screening [15].

High chest radiography prescription rates may still be explained by the absence of national guidelines and it might be guessed that the European Code Against Cancer recommendations [1] do not have any impact on prescription practices. Ignorance of the formal recommendations on the issue might be an explanation, especially in countries without a strong tradition in primary care medicine.

Some limitations should be discussed. First, despite the fact that screening chest radiography is being studied from the sixties and onwards, this is the first study analyzing its practice among the general population. Since the Greek primary care system based on specialized physicians is "newborn", it might be precarious to generalize these findings globally. Second, we analyzed only patients that underwent chest radiography within three years. This may under-estimate the proportion of individuals screened since many of them may have undergone screening chest radiograms in an antecedent date. Furthermore, data were derived from a cross-sectional study on a large convenience sample of the Greek healthy adult population. This design has limited internal validity and is sensitive to a variety of biases. Nevertheless, cross-sectional surveys are most commonly used, and are considered appropriate and easy to perform.

## Conclusion

Chest radiography practice for screening purposes is an old habit that dies hard. More research should be conducted concerning the causes and possible remedies of this phenomenon.

## Competing interests

The author(s) declare that they have no competing interests.

## Authors' contributions

KK was the coordinator of the Greek branch of the study. He was still actively involved in the discussion of the project & study planning, and manuscript writing. GC: statistician, main co-operator from the University of Milan (Italy), dept. statistics, he was still actively involved in the discussion of the project, statistics and manuscript writing. DM: main coordinator of the Italian and Greek braches of the study. He was still actively involved in the discussion of the project, realization of the draft & study planning, review of data abstraction and manuscript writing. VL was involved in study planning and he was responsible for data collection in Peloponnesus and Cephalonia island. He was still actively involved in the discussion of the project and manuscript. IC: statistician, second co-operator from the University of Milan (Italy), dept. Statistics, he was still actively involved in the discussion of the project, statistics and manuscript discussion. AX: responsible for double-blind controls of data inserted by data-managers (thus allowing data-feasibility for both hard- and electronic-data). He was still actively involved in the discussion of the project & study planning, and manuscript writing. CP: main data-manager. She was involved in the study planning and was responsible for data collection in the north-western part of Greece. She was responsible of data entering in the peripheral units of SESy database. She was involved in manuscript discussion. VG was actively involved in the discussion of the project, realization of the draft, discussion of the outcomes, reviewing and formatting the manuscript. AM was involved in the study planning and was responsible for data collection in north-eastern Greece. He was still actively involved in the discussion of the project and manuscript. DK was involved in the study planning and was responsible for data collection in north-central Greece. He was still actively involved in the discussion of the project and manuscript. GZ was involved in the study planning and was responsible for data collection in the wide area of Attika. Hhe was still actively involved in the discussion of the project and manuscript. IK was involved in the study planning and was responsible for data collection in Athens area and the province of Kozani. She was still actively involved in the discussion of the project and manuscript. GF: PACMeR internal statistician. He was involved in study planning, definition of outcome, and draft writing. He constitutes a basic internal support (Greek branch of the study) that avoids possible miss-understanding in the international collaboration. He still participated in the discussion of the results. AP was involved in the study planning and was responsible for data collection in Piraeus area. He was still actively involved in the discussion (any study phase) since his area of expertising is respiratory-diseases. All authors read and approved the final manuscript.

## Supplementary note

Exact phrasing of the tobacco and chest radiography related questions used for both males and females during the questionnaire-based interviews

Tobacco-related questions:

Are You a smoker? [No] [yes]

How old did you start smoking? []

How old did you stop smoking? []

How many cigarettes/tobacco do you daily smoke?......................................................

Chest radiography related questions:

When did you perform chest radiography last time?

ϒ never ϒ Within 1 year ϒ 2 years ϒ 3 years ϒ 5 years ϒ more than 5 years

For which reason did you do it?.....................................................................................

At what frequency do you undergo chest radiography?.................................................

## Pre-publication history

The pre-publication history for this paper can be accessed here:



## Figures and Tables

**Table 1:** Characteristics of the surveyed population

	**N**	**%**
***Gender***		
male	2551	46.4
female	2948	53.6
		
***Age (years)***		
<45	478	8.8
45–64	2830	51.6
65–74	1591	29.0
≥75	586	10.7
		
***Profession***		
Farmers	985	18.4
Craftsmen	425	8.0
Clerks and employees	1053	19.7
Scientists	129	2.4
Freelance prof.	479	9.0
Pensioners	756	14.1
Housewives	1432	26.8
Security Forces	63	1.2
Other labor conditions	20	0.4
		
***Smoking Activity***		
No smokers	3227	59.5
smokers	1626	30.0
Ex smokers	570	10.51
		
***Number of daily cigarettes***		
< 10	416	7.7
10–19	859	16.0
20–29	181	3.4
30–39	404	7.5
≥ 40	225	4.2
None	3301	61.2
		
***Duration of smoke***		
< 10 years	52	2.5
10–19 years	205	9.8
20–29 years	518	24.7
30–39 years	601	28.6
≥ 40 years	723	34.4

Table legend: N = absolute number of responder for each category; % = percentage of individuals per each category. For the evaluation of the following two subgroups (1) number of daily cigarettes and (2) duration of smoke, both smokers and ex-smokers were included in the calculation.

**Table 2:** Individuals who underwent chest radiography for screening purposes within 1, 2, 3 and 5 years.

	**Males (N = 2551)**		**Females (N = 2948)**	
	**Absolute %**	**Cumulative %**	**Absolute %**	**Cumulative %**
**1 year**	12.3	12,3	11.7	11.7
**2 years**	4.6	16.9	4.0	15.7
**3 years**	4.1	21.0	3.3	19.0
**5 years**	3.3	24.3	4.7	23.7

Table legend: N = absolute number of responder for each category; % = percentage of individuals per each category.

**Table 3:** Frequencies by which the test is performed among 936 individuals who did chest radiography for screening purposes within 3 years.

**Frequency**	**N**	**%**
**≤ 6 m**	37	3.95
**7–12 m**	189	20.19
**11–24 m**	163	17.41
**25–36 m**	60	6.41
**37 m – 5 y**	179	19.12
**> 5 y**	2	0.21
Not performed at fixed intervals	111	11.86
Upon medical recommendation	56	5.98
Generally don't do it	139	14.85

**Legend: **N = absolute number of responder for each category; (%) = percentage of individuals per each category; m = months; y = years.

**Table 4:** Probability to undergo chest radiograms for screening purposes: logistic regression for univariate analyses.

	**OR**	**95% CI**	
***Gender***			
Females	1.		
Males	1.130	0.988	1.292
			
***Age (years)***			
< 45	1.		
45–64	1.040	0.809	1.337
65–74	1.158	0.891	1.505
≥ 75	0.923	0.674	1.264
			
***Profession (Prof.)***			
Other professions	1.		
Profession at risk for lung diseases	0.846	0.713	1.003
Pensioners	1.319	1.093	1.593
			
***Smoking Activity***			
No smokers	1.		
Ex smokers	0.894	0.710	1.125
Smokers	1.053	0.907	1.223
			
***Number of daily cigarettes***			
None	1.		
1–19	0.935	0.767	1.140
≥ 20	1.002	0.851	1.179
			
***Duration of smoke***			
<10 years	1.		
10–19 years	1.135	0.539	2.387
≥ 20 years	0.871	0.442	1.719
			
***Family history for lung cancer***			
No	1.		
Yes	1.251	0.988	1.583
			
***Family history for malignancies***			
No	1.		
Yes	0.929	0.806	1.071

**Table legend: **OR = Odds Ratios; CI = Wald Confidence Interval

**Table 5:** Probability to undergo chest radiograms for screening purposes: multivariate logistic regression

	**OR**	**95% CI**	
***Gender***			
Females	1.		
Males	1.188	1.013	1.393
			
***Age (years)***			
< 45	1.		
45–64	0.986	0.760	1.280
65–74	1.045	0.787	1.388
≥ 75	0.817	0.581	1.147
			
***Profession (Prof.)***			
Other professions	1.		
Profession at risk for lung diseases	0.808	0.675	0.968
Pensioners	1.277	1.041	1.568
			
***Smoking Activity***			
No smokers	1.		
Ex smokers	0.863	0.675	1.103
Smokers	1.022	0.868	1.203
			
***Family history for lung cancer***			
No	1.		
Yes	1.200	0.942	1.529

**Table legend: **OR = Odds Ratios; CI = Wald Confidence Interval.

## References

[B1] Boyle P, Autier P, Bartelink H, Baselga J, Boffeta P, Burn J, Burns HJ, Christensen L, Denis L, Dicato M, Diehl V, Doll R, Franceschi S, Gillis CR, Gray N, Griciute L, Hackshaw A, Kasler M, Kogevinas M, Kvinnsland S, La Vecchia C, Levi F, McVie JG, Maisonneuve P, Martin-Moreno JM, Bishop JN, Oleari F, Perrin P, Quinn M, Richards M, Ringborg U, Scully C, Siracka E, Storm H, Tubiana M, Tursz T, Veronesi U, Wald N, Weber W, Zaridze DG, Zatonski W, zur Hausen H (2003). European Code Against Cancer and scientific justification: third version. Ann Oncol.

[B2] (2004). US Preventive Services Task Force Lung Cancer Screening: Recommendation Statement. Annals Inter Med.

[B3] National Cancer Institute Screening for lung cancer (PDQ). http://www.cancer.gov/cancerinfo/pdq/screening/lung/healthprofessional/.

[B4] Smith RA, Mettlin CJ, Davis KJ, Eyre H (2005). American Cancer Society guidelines for the early detection of cancer. CA Cancer J Clin.

[B5] Morrison BJ Interventions other smoking cessation to prevent lung cancer. Update for the Canadian Task Force on preventive health care. http://www.ctfphc.org/Abstracts_printable/Ch64abs.htm.

[B6] Humphrey LL, Teutsch S, Johnson M, U.S. Preventive Services Task Force (2004). Lung cancer screening with sputum cytologic examination, chest radiography, and computed tomography: an update for the U.S. Preventive Services Task Force. Ann Intern Med.

[B7] Manser RL, Irving LB, Stone C, Byrnes G, Abramson M, Campbell D (2004). Screening for lung cancer. Cochrane Database Syst Rev.

[B8] Sladden MJ, Ward DoJE (2004). Australian family physicians screen smokers for lung cancer?. Chest.

[B9] Ashford A, Gemson D, Sheinfeld Gorin SN, Bloch S, Lantigua R, Ahsan H, Neugut AI (2000). Cancer screening and prevention practices of inner-city physicians. Am J Prev Med.

[B10] Smith HE, Herbert CP (2003). Preventive practice among primary care physicians in British Columbia: relation to recommendations of the Canadian Task Force on the Periodic Health Examination. CMAJ.

[B11] Beaulieu MD, Rivard M, Hudon E, Beaudoin C, Saucier D, Remondin M (2002). Comparative trial of a short workshop designed to enhance appropriate use of screening tests by family physicians. CMAJ.

[B12] Nakar S, Vinker S, Neuman S, Kitai E, Yaphe J (2002). Baseline tests or screening: what tests do family physicians order routinely on their healthy patients?. J Med Screen.

[B13] Lynch GR, Prout MN (1986). Screening for cancer by residents in an internal medicine program. J Med Educ.

[B14] (1990). ACS 1989 survey of physicians' attitudesand practices in early cancer detection. CA Cancer J Clin.

[B15] Proiskos A, Loukidou E, Kamposioras K, Bristianou M, Zina V, Pliadi O, Karakatsanis A, Alexiou G, Gkougkoutsi A, Mauri D (2005). Screening chest radiography in primary care: an underestimate belief. Eur J Gen Pract.

[B16] Montano DE, Phillips WR (2005). Cancer screening by primary care physicians: a comparison of rates obtained from physician self-report, patient survey, and chart audit. Am J Public Health.

[B17] Woodward CA, Hutchison BG, Abelson J, Norman G (1996). Do female primary care physicians practise preventive care differently from their male colleagues?. Can Fam Physician.

[B18] Hutchison B, Woodward CA, Norman GR, Abelson J, Brown JA (1998). Provision of preventive care to unannounced standardized patients. CMAJ.

[B19] Mauri D, Pazarlis P, Mauri J, Altinoz H, Rivas Flores FJ, Karentzou I, Proiskos A, Lakiotis V, Maragkaki A, Terzoudi E, Dambrosio EM, Spiliopoulou A, Varsami A, Alexandropoulou P, Tolis C, Pavlidis N, Vittoraki A (2004). Sesy Europe: a multi-language database dedicated to cancer screening monitoring. J Exp Clin Cancer Res.

[B20] Mauri J, Mauri D, Pazarlis P, Altinoz H, Rivas Flores FJ, Karentzou I, Priskos A, Lakiotis V, Alevizaki P, Terzoudi E, Dambrosio M, Spiliopoulou A, Alexandropoulou P, Kalogerakis D, Varsami A (2004). PC 3 component database for community-based medical trials. A cost-effective solution for both voluntary associations and institutions of the Emerging World. Gazz Med Ital – Arch Sci Med.

[B21] The SAS Package. http://www.dcs.napier.ac.uk/peas/saspackage.htm.

[B22] Gray JA (2004). New concepts in screening. Br J Gen Pract.

[B23] Diederich S, Lenzen H (2000). Radiation exposure associated with imaging of the chest: comparison of different radiographic and computed tomography techniques. Cancer.

[B24] Kubik A, Polak J (1986). Lung cancer detection. Results of arandomized prospective study in Czechoslovakia. Cancer.

[B25] Frost JK, Ball WC, Levin ML, Tockman MS, Baker RR, Carter D, Eggleston JC, Erozan YS, Gupta PK, Khouri NF (1984). Early lung cancer detection: results of the initial (prevalence) radiologic and cytologic screening in the Johns Hopkins study. Am RevRespir Dis.

[B26] Karakatsanis A, Alexiou G, Kalogerakis D, Mauri D Health economics: is smoking a trigger point for screening chest radiography opportunistic practice?. Can Med Ass J (e-letter).

